# Perspectives of old-age and dementia researchers on communication with policymakers and public research funding decision-makers: an international cross-sectional survey

**DOI:** 10.3389/fmed.2024.1472479

**Published:** 2024-12-20

**Authors:** Peter Fusdahl, Miguel Germán Borda, Jonathan Patricio Baldera, Dag Aarsland, Ara Khachaturian, Geir Sverre Braut

**Affiliations:** ^1^Centre for Age-Related Medicine, Stavanger University Hospital, Stavanger, Norway; ^2^Faculty of Medicine, University of Bergen, Bergen, Norway; ^3^Department of Neurology, Clínica Universidad de Navarra, Pamplona, Spain; ^4^Centro de Investigación en Ciencias de la Salud, Facultad de Ciencias de la Salud, Universidad Anáhuac Mexico, Huixquilucan, Edo De Mexico, Mexico; ^5^Department of Old Age Psychiatry, Institute of Psychiatry, Psychology, and Neuroscience, King’s College London, London, United Kingdom; ^6^Campaign to Prevent Alzheimer’s Disease, Potomac, MD, United States; ^7^Brain Watch Coalition of the Campaign to Prevent Alzheimer’s Disease, Potomac, MD, United States; ^8^International Neurodegenerative Disease Research Center, International Neurodegenerative Disorders Research Center, Prague, Czechia; ^9^National Supercomputing Institute and Dedicated Research Network, University of Nevada, Las Vegas, NV, United States; ^10^Western Norway University of Applied Sciences, Bergen, Norway; ^11^Department of Research, Stavanger University Hospital, Bergen, Norway

**Keywords:** Alzheimer’s disease, old age medicine, brain health, public health care research, health care management, dementia, research funding

## Abstract

**Introduction:**

Society commonly believes that research knowledge is complementary to public decision-making. This study aimed to understand the perspectives and implications of dementia researchers communicating with policymakers and public research decision-makers (public officials).

**Methods:**

This study uses 24 questions from an anonymous, online survey, which was received by 392 members of nine European, Latin American, and United States medical researcher associations/networks in the fields of age-related neurological degeneration and dementia medicine. The data were analyzed via crosstab analysis, two group comparison analyses, and a logistic regression model.

**Results:**

In total, 91 (23.2%) respondents completed the questionnaire. Eight independent variables were related to researchers’ research discipline, research remuneration, experience, authorship, H-index, and research grants. The statistically significant variables determining whether the respondents had contact with public officials were “Years of research experience” (*p* = 0.004), “Number of articles first-authored in the last 5 years” (*p* = 0.007), and “Average H-index in the last 5 years” (*p* = 0.048) [median (IQR)]; 47% of the surveyed researchers had been in contact with public officials in the last 12 months. The most frequently communicated topics were the importance of their own research to society (61%) and their own funding (60%); 87% (*n* = 79) of the researchers did not believe that public officials had a very good understanding of their dementia research.

**Conclusion:**

Less than half (47%) of dementia researchers communicate with public officials, and they communicate mostly about the importance of their own research and funding their own research. Nine of 10 researchers do not believe that public officials understand their research well.

## 1 Introduction

It has been a belief for centuries that research and knowledge communication are complementary to policymaking (1), and researchers should be required to communicate with stakeholders outside the research community (2). In Europe, the European Commission introduced responsible research and innovation (RRI) as an administrative framework to promote research and innovation activities to contribute to societal wellbeing (3, 4).

The communication of medical research knowledge to policymakers and public decision-makers (“public officials”) is complicated and complex. Many studies address how to bridge the knowledge gap between researchers and policymakers (5), and these provide detailed descriptions of how researchers should adapt communication to the public stakeholders. However, a literature search revealed little relevant research on the medical researchers’ perspective on funding communication with these public funders and how it works.

Dementia diseases are the leading cause of death in the United Kingdom (6), and the 7th top cause of death in the world (7); however, more importantly, dementia is one of the largest cost drivers for morbidity and loss of personal autonomy (8–10). Dementia is the only top 10 cause of death with no prevention or cure, yet it is among the most expensive conditions to manage. Despite recent regulatory approvals, 40 years of dementia research have not resulted in any safe, effective, and affordable prevention or treatment interventions (11). Dementia diseases have higher medical and social costs than cancer and heart/lung diseases. We expect an exponentially growing population with mild cognitive impairment and dementia (12), but dementia research and care remain chronically underfunded (7).

There is no common understanding of what is considered evidential knowledge in public policymaking decisions (13). Many stakeholders are involved in policymaking and public research funding decisions, and these stakeholders’ behavior changes dynamically in shifting political and societal contexts (14). Furthermore, there are no common standards for defining public health needs or research health returns (13, 15). This adds complexity to the common understanding of the researcher’s role and challenges the complementary fit of scientific knowledge, public health needs, and the policymaker’s ultimate allocation of research funding (13, 16, 17).

The purpose of this study was to explore the degree and content of contact with public stakeholders and policymakers by international dementia researchers and how this differs depending on researchers’ demographics and past funding success. This information provides a baseline for future actions to improve dementia researchers’ substantive contributions to the societal wellbeing of a growing population with mild cognitive impairment and dementia.

## 2 Methods

An anonymous cross-sectional online survey was developed to provide quantitative descriptive data.

### 2.1 Survey design

An academic online survey platform, *nettskjema.no*, was utilized for data collection. *Nettskjema.no* is specifically designed to gather sensitive data securely and confidentially, with built-in functionality for conducting anonymous surveys.

A rigorous multistep process was employed to assess the validity of the questionnaire with respect to offering meaningful data, repeatability of the results, and fit for the specific purpose (18). This included a validation process generating six revisions before the final survey versions were written in English and Spanish.

The questionnaire was designed so that all questions were required to be answered before the answers were submitted. An ethical concern related to such forced answers (19) was mitigated by allowing respondents to also answer “I do not know” or “I prefer not to answer” and options to answer “Other” in text boxes for multiple-choice questions. These answer options were also included considering the clarity of questions, familiar vocabulary, sequence of questions, and avoidance of double-barreled questions (20). Sex and age were not included in the descriptive questions to ensure anonymity and to increase the respondents’ confidence in survey anonymity.

The final questionnaire had 72 questions including skip questions depending on previous answers. The questions included multiple-choice questions, with follow-up questions if the standard questions did not fit, numerical fill-in boxes, and short answer questions.

### 2.2 Validation

The questionnaire was developed through a validation process in which multiple validities were tested (21, 22).

#### 2.2.1 Face and content validity

The questions and survey topics in the survey were outlined through an anticipatory review to obtain an overall understanding of how the questions reasonably appeared to obtain the data needed. A literature search was also conducted to identify available and relevant questionnaires and/or questions, but the search did not yield applicable results. The first revision of the questionnaire was reviewed for content validity. Face and content validity, hereunder relevant components and sub-traits, were reviewed qualitatively by four professors in age-related and dementia research, who are experienced public health managers with extensive research funding experience. The second revision was also reviewed for face and content validity by three professors in social sciences and business management.

#### 2.2.2 Internal, language, and cultural validity

The aim of the questionnaire was to explore perceptions and views held by dementia researchers. Thus, validity testing of “the correctness of inferences about causal connections between two focal constructs” (23) was not included. The questionnaire was intended for English/Spanish-speaking researchers working in Europe, the United States, and Latin America. Two focus groups reviewed the third version in an international cultural context. The two focus groups each had four members consisting of PhD fellows and postdocs in dementia research, with European and Latin American members, respectively. A fourth revision was translated into Spanish by an experienced age-related and dementia researcher, who has Spanish as the mother tongue. Another experienced dementia researcher, who did not participate in the English-to-Spanish translation, translated the Spanish version back to English text. The original English fourth revision and the “translated and back” English–Spanish–English version were then compared to develop an English and Spanish fifth revision with language validity. The fifth revision was reviewed by Stavanger University Hospital’s Data Protection Officer, *eProtocol,* refer Chapter 9.6 Ethical considerations, to ensure respondent anonymity, and revised accordingly for a sixth revision.

#### 2.2.3 External validity

External validity of the questionnaire was addressed by conducting a pilot test of the English and Spanish questionnaires (version six). The pilot test included 100 English and 38 Spanish-speaking clinical brain researchers (PhD fellows and postdocs) in Europe, Latin America, and North America. The pilot test respondents were not included in the respondent group for the survey. The questionnaire used in the pilot testing included a free text question, allowing the pilot test respondents to comment on the questionnaire. Twenty-five respondents answered the pilot test questionnaire. Two technical issues related to skip questions were identified and corrected for the sixth revision of the questionnaire used in the survey.

#### 2.2.4 Construct validity

The questionnaire explored perceptions in an area of interest where the theoretical and empirical background knowledge is limited and unclear, and the results were not intended to be compared with preestablished psychometrics. Thus, such construct validity has not been deemed appropriate by the authors (24, 25).

### 2.3 Sample and sampling

The survey was sent to 433 active clinical researchers listed as members of nine national medical associations and national research networks in Europe, Latin America, and the United States in age-related neurological degeneration and dementia medicine. The authors did not have direct access to the invited clinical researchers but were reached through the respective associations and network contacts. The age-related neurological and dementia medicine community collaborates across nations and was considered relatively transparent, and the associations and research networks were identified by the authors. The authors drafted the invitations sent by the chairperson/leading representative of each association/network to its respective members.

### 2.4 Data collection

The invitation email to participate included an invitation text that stated the following:

- The survey was anonymous.
- The purpose of the survey was to better understand how medical researchers perceive and experience contact with policymakers and decision-makers in public funding institutions in Europe, Latin America, and the United States.
- This survey focused on the researcher’s perspective, not just the perceptions of funding institutions.
- The anonymous responses and the respondent data would be stored for a maximum of 12 months in a facility approved for strictly confidential health research data in Norway.^1^
- The respondents could use any device, such as a computer, tablet, or mobile.
- The questions were quick and easy to answer in approximately 10–15 min.
- The link to the online survey was provided.

The initial invitation was followed by two reminders during the 11-week open response period.

### 2.5 Data analysis

The data recorded were analyzed by sorting each respondent’s answers with respect to the 13 survey questions dedicated to this study and the descriptive data in a cross-tabulation table (26): Highest level of education, main research discipline, employer(s), main working area, paid to research or not, average years of research experience, average number of articles written as first author in the last 5 years, average number of articles as author in the last 5 years, average H-index in the last 5 years, average numbers of grant applications in the last 36 months, and average numbers of grants awarded in the last 36 months.

A descriptive analysis was conducted on the variables included in the analysis. This included calculated frequencies for categorical variables and means for continuous variables, in addition to their respective standard deviations and medians with interquartile ranges for continuous variables. Two group comparison analyses were performed to assess differences by “researchers’ primary working area” and “researchers been in contact and dialog, or not, with policymakers or public decision-makers to discuss issues related to research” with respect to the variables included in the study. Finally, logistic regression models were fitted to explore the associations between “researchers been in contact and dialog, or not, with policymakers or public decision-makers to discuss issues related to research” and the variables listed above. For illustration purposes, two figures were developed to present (1) the topics discussed with the policymakers and political decision-makers and (2) the reasons for not being in contact and dialog with policymakers and political decision-makers. We used a significance level for type 1 error of 0.05. R software version 4.3.1 was used to carry out the statistical analyses.

## 3 Results

Forty-one of the 433 invitation emails were reported not to work and/or “bounce,” and 392 researchers were registered as contacted. A total of 91 survey responses were received (response rate 23.2%). See Table 1 for respondent demographics.

**Table 1:** Respondent demographics.

Respondent demographics		
*N* = 91		
	*n*	%
Highest education		
Medical school	4	4%
Medical specialist	37	41%
PhD	45	49%
Other	5	5%
	91	100%
Primary working area		
Europe	46	51%
Latin America	30	33%
United States/Canada	12	13%
Other	3	3%
	91	100%
Main research discipline		
Clinical	82	90%
Basic science	9	10%
	91	100%
Responders employer(s)		
University hospital	29	32%
University hospital + university	22	24%
University	20	22%
Not specified*	6	7%
Private research center/company	4	4%
Hospital + private research center/company	3	3%
University + hospital + private research center/company	4	4%
Private + not specified*	1	1%
University + not specified*	2	2%
	91	100%

*Not specified, Respondent options: “Other” or “I prefer nor to answer”.

The old age and dementia researcher included in the study had significant differences (*p* = 0.011), with European/US/Canadian responders having PhD education 59% (*n* = 36), compared to 30% (*n* = 9) in Latin America; 60% of the Latin American respondents were medical specialists. The respondents reported that most of their research collaborations were in their primary working area, but with more international collaboration by the European/US/Canadian respondents. European/US/Canadian respondents collaborated 85 and 62% with European and US/Canadian peers, respectively. Latin American respondents collaborated 93 and 37% with Latin American and European researchers, respectively (*p* < 0.001). The research discipline (clinical or base research) was not significantly different between the two regions. A significantly (*p* = 0.007) higher share of European/US/Canadian respondents (74%, *n* = 45) worked for a university hospital, compared to their respondents in Latin America (43%, *n* = 13), who had significantly more private employment with private research centers/companies (17%, *n* = 5, *p* = 0.042). See Table 2.

**Table 2:** Respondents’ characteristics by primary working area.

Variables	Region for primary working area		Overall *n* = 91 (100%)	*p*- value
	Europe/USA/Canada *n* = 61 (67.1%)	Latin America *n* = 30 (32.9%)		
Highest level of formal education, (%)				
Graduate medical school	4 (6.6%)	0 (0.0%)	4 (4.4%)	**0.011**
Medical specialist	19 (31.1%)	18 (60.0%)	37 (40.7%)	
PhD	36 (59.0%)	9 (30.0%)	45 (49.5%)	
Other	2 (3.3%)	3 (10.0%)	5 (5.5%)	
Researchers collaborated from, (%)				
Latin America and the Caribbean	11 (18.0%)	28 (93.3%)	39 (42.9%)	**< 0.001**
Europe	52 (85.2%)	11 (36.7%)	63 (69.2%)	
USA/Canada	38 (62.3%)	7 (23.3%)	45 (49.5%)	
Australia	17 (27.9%)	0 (0.0%)	17 (18.7%)	
Asia	16 (26.2%)	0 (0.0%)	16 (17.6%)	
Other regions	4 (6.6%)	1 (3.3%)	5 (5.5%)	
Main research discipline, (%)				
Clinical	54 (88.5%)	28 (93.3%)	82 (90.1%)	0.727
Basic science	7 (11.5%)	2 (6.7%)	9 (9.9%)	
Respondent employer(s), (%)*				
University/college	30 (49.2%)	16 (53.3%)	46 (50.5%)	0.714
University hospital	45 (73.8%)	13 (43.3%)	58 (63.7%)	**0.007**
Public research center	4 (6.6%)	4 (13.3%)	8 (8.8%)	0.343
Private research center/company	1 (1.6%)	5 (16.7%)	6 (6.6%)	**0.042**

*Multiple answer questions. For *p*- value, we have estimated the differences in each response. Bold *p*-values indicate statistically significant variables.

Forty-three (47%) of the active dementia researchers had been in contact with policymakers and public decision-makers in the last 12 months. These researchers are experienced researchers with 17.7 years of experience and an H-index of 31. Table 3 shows that 47 (52%) dementia researchers stated that they had not been in contact with these public officials, and they have on average 5.7 years less (12.0 years) research experience and an H-index of 19. The average years of research experience show a significant association with engagement with policymakers and public decision-makers (mean (SD) *p*-value = 0.005; median (IQR) *p* = 0.004).

**Table 3:** Differences in researchers being in contact with policymakers and public decision-makers—or not.

Variables	Been in contact and dialog with policy or public decision-makers to discussed issues related to research *		Overall	*p*- value
	No	Yes		
	(*n* = 47; 51.6%)	(*n* = 43; 47.3%)	(*n* = 91; 100.0%)	
Highest level of formal education, (%)				
Graduate medical school	3 (6.4%)	1 (2.3%)	4 (4.4%)	0.567
Medical specialist	16 (34.0%)	20 (46.5%)	37 (40.7%)	
PhD	25 (53.2%)	20 (46.5%)	45 (49.5%)	
Other	3 (6.4%)	2 (4.7%)	5 (5.5%)	
Main research discipline, (%)				
Clinical	44 (93.6%)	37 (86.0%)	82 (90.1%)	0.232
Basic science	3 (6.4%)	6 (14.0%)	9 (9.9%)	
Region for primary working area				
Latin America and the Caribbean	12 (25.5%)	18 (41.9%)	30 (33.0%)	0.101
Europe/USA/Canada	35 (74.5%)	25 (58.1%)	61 (67.0%)	
Researcher paid to research**				
Yes	19 (40.4%)	14 (33.3%)	33 (36.7%)	0.489
No	28 (59.6%)	28 (66.7%)	57 (63.3%)	
Average years of research experience, mean (SD)				
Mean (SD)	12.0 (7.47)	17.7 (10.8)	15.1 (10.2)	**0.005**
Median (IQR)	12 (6, 15)	17 (10, 24.5)	14 (8, 20)	**0.004**
Average no. of article as first author last 5 years, mean (SD)				
Mean (SD)	8.98 (24.9)	16.6 (21.5)	12.5 (23.4)	0.125
Median (IQR)	4 (2, 7)	9 (3, 23)	5 (2, 10)	**0.007**
Average no. of article as author last 5 years, mean (SD)				
Mean (SD)	62.7 (150)	117 (252)	90.6 (206)	0.220
Median (IQR)	20 (8, 50)	45 (10, 95)	31 (10, 80)	0.070
Average H-index last 5 years				
Mean (SD)	18.7 (25.5)	30.8 (34.3)	25.2 (31.1)	0.063
Median (IQR)	11 (2, 27)	19 (5, 46)	16 (2.5, 33.5)	**0.048**
Average no. of grants participated last 36 months				
Mean (SD)	6.47 (7.38)	8.58 (11.1)	7.95 (10.3)	0.296
Median (IQR)	4 (0.5, 10)	5 (3, 9)	5 (2, 10)	0.231
Average no. of grants awarded last 36 months				
Mean (SD)	3.23 (4.33)	3.74 (4.44)	3.88 (5.79)	0.583
Median (IQR)	2 (0, 4)	3 (1, 4)	2 (1, 4)	0.143

*Response “I prefer not to answer” is omitted to ensure the anonymity of the respondent (*n* = 1). **Was excluded “Not applicable option”. Bold *p*-values indicate statistically significant variables.

Those who had been in contact with policymakers had a mean of 16.6 first-author articles, whereas those who had not had a mean of 9.0 articles. The median and interquartile range (IQR) (*p*-value of 0.007) indicate that this difference might be significant and suggest a potential trend toward a greater number of first-author publications among those who engage with policymakers; however, the mean (SD) (*p* = 0.125) indicated that the difference is not significant.

The variable for H-Index for the last 5 years revealed that respondents who engaged with policymakers had a higher mean H-Index (30.8) than those who did not (18.7), with a *p*-value of 0.063, indicating a difference with borderline significance. The median (IQR) H-index values (11 vs. 19) suggest that a higher H-Index might be associated with greater policy engagement.

The results revealed no significant differences in the respondents’ contact and dialog based on the degree of formal education, research discipline, whether they were paid to research, number of coauthored articles, or grant applications/awards.

A logistical regression suggests two statistically significant variables determining old age and dementia researcher contact with policymakers and public decision-makers—or not. “Years of research experience” and “H-index last 5 years.” The remaining variables do not demonstrate significant associations with the dependent variable, hereunder “number of articles written as first author” and “number of grant applications participated in last 36 months,” see Table 4.

**Table 4:** Modeling old-age and dementia researchers’ contact with policymakers and public decision-makers.

Dependent variable: The researcher has been in contact and dialog with policy or public decision-makers to discuss issues related to research				
Variables	OR	CI 95%		*p*- value
		Lower	Upper	
Main research discipline, (%)**				
Clinical	1.000			
Basic science	2.691	0.646	13.746	0.190
Researcher paid to research**				
Yes	1.000			
No	2.152	0.808	6.250	0.138
Years of research experience**	1.097	1.037	1.173	**0.003**
No. of article as first author last 5 years**	1.024	1.001	1.059	0.094
No. of article as author last 5 years**	1.002	1.000	1.006	0.191
H-index last 5 years**	1.024	1.006	1.047	**0.020**
No. of grants participated in last 36 months**	1.055	0.998	1.132	0.093
No. of grants awarded in last 36 months*	1.066	0.958	1.200	0.257

*Adjusted by region. **Adjusted by region and education. Bold *p*-values indicate statistically significant variables.

About half of the researchers (49%, *n* = 21) who were in contact with public officials in the last 12 months did so only 1–2 times; 14% of these researchers (*n* = 6) were in contact 7+ times, and these researchers had authored 3.6 times (241 articles vs. 66 articles) more scientific articles than the former group and a 2.2 times higher H-index at 48.

**Figure 1:**
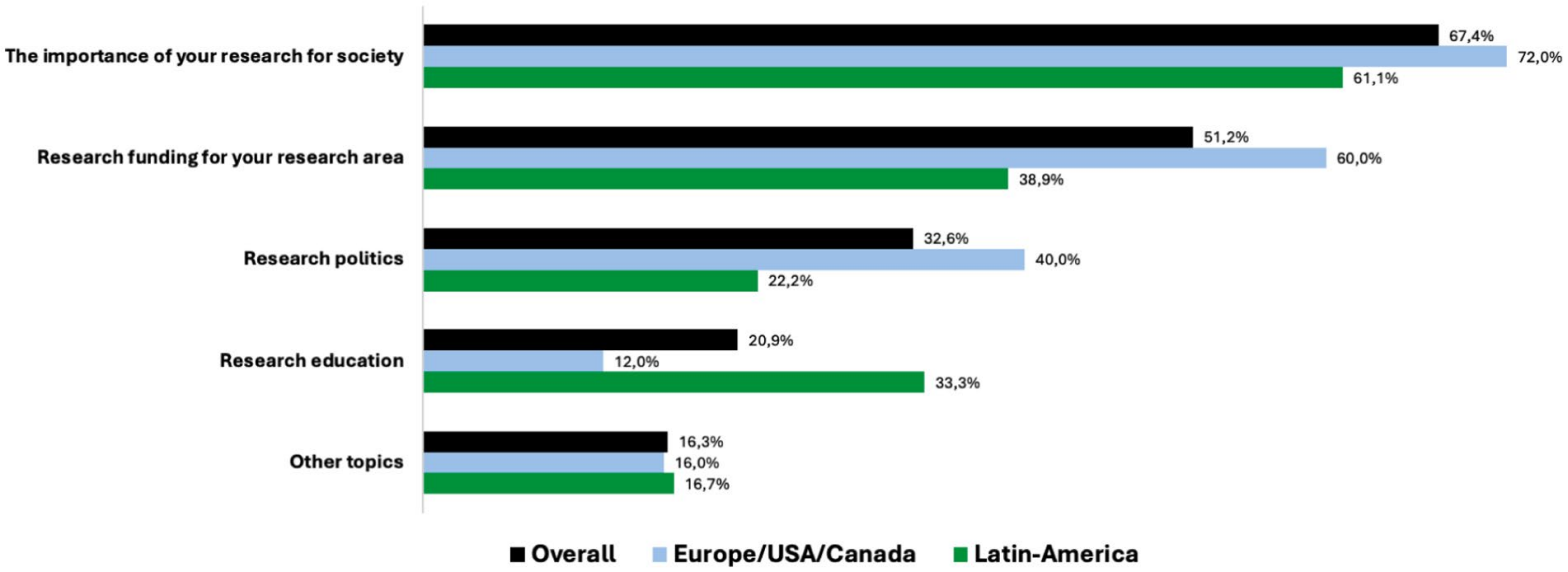
Topics discussed with the policymakers and political decision-makers (answers in % of respondents).

The importance of the researcher’s own research for society (36%, *n* = 29) and the need for funding own research (27%, *n* = 22) were the two most reported discussion topics with the public officials. 17% (*n* = 14) of the researchers reported that they discussed research politics with the public officials (Figure 1).

**Figure 2:**
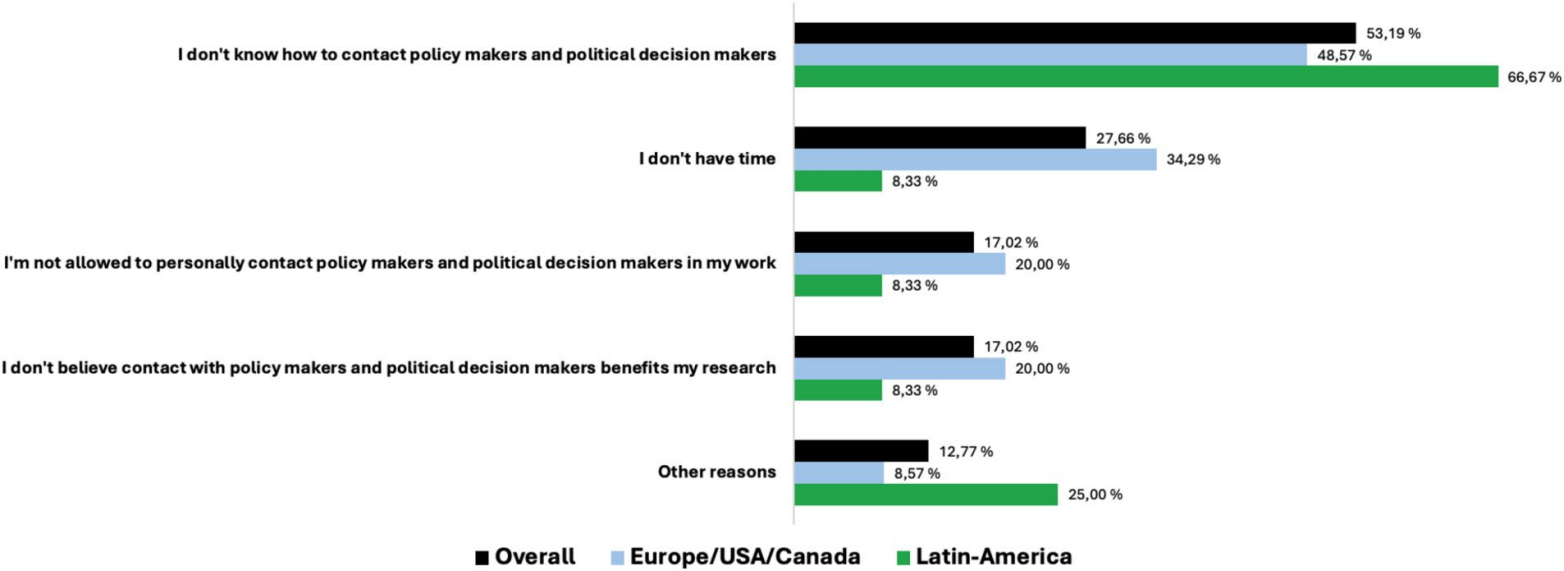
Reasons for not being in contact and dialog with policymakers and political decision-makers (answers in % of respondents).

The top three reasons for researchers not contacting public officials were (1) that they did not know how to do it (42%, *n* = 25), (2) they did not have time (22%, *n* = 13), or (3) they did not think it will benefit own research (13%, *n* = 8) (Figure 2).

Regardless of whether or not they had had contact with public officials, 87% (*n* = 79) of the respondents did not believe politicians or policymakers had a very good understanding of their dementia research; 10% (*n* = 9) of the researchers disagreed and thought politicians and policymakers had a very good understanding of their dementia research. It can be noted that these researchers were awarded 74% of the grants they applied for over the last 36 months (“grant application margin”). The same number of researchers (87%, *n* = 79) continued to believe it would be useful to discuss the alignment of political and research objectives; 87% (*n* = 73) of the researchers who were positive about discussing such alignment of objectives were also willing to use their own time for such dialog. Again a minority of researcher did not think such alignment of political and research objectives would be useful (5%, *n* = 5), and 8% (*n* = 7) would neither be willing to use own time to do it. The minority of researcher not willing to use own time to align objectives with the politicians reported that they were awarded 70% of the grants they applied for.

Ninety-five percent (*n* = 86) of dementia researchers with a PhD and medical background believed that new and novel research ideas are needed in their dementia research area. The 12% (*n* = 11) of researchers who believe that public officials are willing to fund new and novel research reports have an average H-index of 52, 312 authored articles, and 23 years of research experience. This contrasts with 57% (*n* = 52) of the researchers, who said that new and novel research only sometimes gets publicly funded, and these researchers have an average of 6 years less research experience and an H-index 30 points lower, i.e., 16 years of experience and an H-index of 22.

Forty-five percent (*n* = 39) of the researchers have received public funding for new and novel research ideas. These funded researchers were a more productive and a more cited group than the researchers who had not received funding for new and novel research, with a 3.3 times higher H-index (37 vs. 12) and 2.9 times more authored articles (128 vs. 43 articles).

A total of 74% (*n* = 59) of the researchers stated that they do have new and novel research ideas that they would like to fund, whereas 14% [(*n* = 11) did not have such new ideas. In particular, the researchers with new and novel research ideas to fund had an average H-index of 13 and 26 articles authored in the last 5 years, while the researcher with no new and novel ideas reported a significantly higher average H-index and number of articles authored in the last 5 years, i.e., average H-index of 21 and 63 articles].

Fifty-six percent (*n* = 45) of the researchers who reported their own funding track-records of new and novel research ideas, thought that too much of the research funding went to established research ideas. These researchers were awarded 42% of the grants they applied for in the last 36 months, which is slightly lower than the 48% grant allocation margin for the researchers who did not think that established research ideas received too much funding.

## 4 Discussion

Surveys have for many years been used to obtain a better understanding of human behavior and preferences (27). An online survey was considered to be the viable option for this study to reach researchers on the European and American continents due to its speed, cost, and questionnaire flexibility while balancing the risks of bias, ambiguity, over-representation, and fraud (20). Disadvantages of an online survey were considered, such as not allowing for follow-up questions, sharing of the survey with friends and colleagues leading to over-representation, deliberate erroneous responses, and lower response rates and bias (28), particularly for surveys in academic settings and to a professional population (29). Care should also be taken with respect to inferring causal relationships from the data and results presented (30). These methodological disadvantages should be noted and considered when interpreting the study results.

There are many studies on response rates, covering the range from detailed survey design to macroeconomic factors across nations (29, 31, 32). An average response rate for online surveys is 36%. Web-based response rates have an average 12% lower expected response rate than other survey modes do. The concern with lower response rates is that the survey results do not represent the survey population and introduce non-response bias. This is, however, not supported by evidence, and response rates of 5–10% can be reliable if the sample size is greater than 500 and a response rate of 20–25% if the sample size is less than 500 (29, 33). This suggests that the response rate for this study can be reliable at 23.3%.

Dementia researchers are, in general, expected to communicate their research to stakeholders in society (2). However, they appear to be divided into two main groups regarding their communication activity: those who engage in communication and those who do not, with respective percentages of 47 and 52% in this study. This suggests that some researchers in the field of aging and dementia may not have embraced a public policy aimed at communicating with societal stakeholders. When three out of four interviewed researchers (76%) have either “not been in contact” or “only communicated 1–2 times over the last 12 months,” researchers may consider communication with public officials as a fiduciary duty, rather than an effective opportunity to have a genuine dialog with the exchange of ideas and knowledge. 87% of all researchers responding to the questionnaire state that they do not think the politicians and policymakers have a very good understanding of their dementia research, i.e., even the researchers who say that they communicate with the public officials answer that they do not believe that the communication improves the public officials’ understanding of their research.

Policymakers and researchers act on different interests, facts, and narratives. Earlier studies demonstrate that policymaking is an intuitive process, rather than an objective, evidence-based activity (34). There is little evidence of how research is used in policy and public decision-making, but there is evidence that policymakers allocate public research funding based on defined societal needs (13, 16, 17). Even targeted ideas and effective lobbying activities must be communicated so that the message is aligned with three political “streams” to create a temporal “window of opportunity” for change: The specific problem must be recognized/defined, political solutions to the problem must have been developed prior to the communication, and there must be a political/public interest in the issue (35, 36).

Another layer of complexity in public research funding is the globalization of health research. Public health research funding is organized and managed differently around the world, also with respect to who determines the priorities and how funds are allocated (37). Moreover, the researchers and research projects receiving the funds are increasingly more diverse, with a wider range of international participation (38). Advice on how to communicate to policymakers and public funders is vague. The combination of these factors makes it therefore reasonable that less experienced researchers communicate less than the more experienced researchers who are focused on fundraising their research projects as principal investigators (PI). The implementation of required communication from a wider and more representative group of researchers can be considered for future public research grants to help voicing research ideas and experiences of less experienced researchers to a public and institutional audience. An example of this could be to require fund applications to include a work package where research knowledge is communicated to multiple audiences by different research team members (PhD fellows), postdocs, and research leads (principal investigators) and that research project narratives are required developed to fit communication with public officials.

The dissonance between the public policy of dissemination of research knowledge and the empirical know-how that policymaking and public decisions are more intuitive could explain why experienced dementia researchers communicate to generate demand for their own research and optimize their own research funding. This would be in line with dementia researchers also limiting collaboration and networking with other researchers who add monetary or social value to their research (39).

Research funding distribution mechanisms and their impacts on public funding institutions and their respective researchers have been studied in detail (38, 40–44), but policy development and the alignment of objectives throughout the research value chain, from public policy through public funding institutions to the executing researcher are not processes well described (37). This study demonstrates that the researchers welcome a dialog with the policymakers and public funders. Nine of 10 (87%) researchers say it would be useful to align public and personal research objectives, and they are willing to use their time to do it. This represents a potentially valuable source of information and a reduction in information bias. Future studies could elaborate on this by examining the anticipatory view that the resultant information and knowledge flowing to policymakers and public funders are biased toward existing and/or established research ideas. This uncertainty is strengthened by 56% claiming that too much public research funding is going to established research ideas. It is interesting to note that research innovation is present with 74% of the researchers having new and novel research ideas that they would like funded. These innovative researchers are not the most experienced or the most productive, as they have an average 10 years of experience and predominantly work for a university and/or a university hospital, and with 57% fewer articles authored (26.4 vs. 62.5 articles), and a 40% lower H-index (12.7 vs. 21.4) compared to the researchers with no new or novel research ideas.

Surprisingly, 95% of the dementia researchers agreed that new and novel research is needed in their research area. A meager 12% of the researchers believe that the public funders are willing to fund new and novel research, whereas the large majority (75%) believe this only happens sometimes or not. These 12% of researchers represent an elite of researchers with an average 23-year track record, and an average production of 312 coauthored articles, H-index of 52 and grant allocation margin of a very high 57%.

Dementia research have been underfunded over time. Decades of research have not developed medical prevention or treatment of dementia diseases. This study suggests that dementia researchers believe that novel research ideas need to be pursued to prevent and treat of dementia diseases in the future, and the dementia researchers have identified these novel ideas. However, information on these novel ideas is not reaching the public research funders. How can we expect policymakers and public funding decision-makers to prioritize new and novel dementia research without this insight? This situation suggests a need for a strategic change in how medical research projects are awarded public research funding. Further study is needed to consider how funding for such new and novel research should be made available, but it could include funding to less experienced researched teams, higher risk project plans, and more cross-discipline research teams.

An alternative public research funding strategy could be to implement a version of the “barbell strategy” from the financial markets (45), where the public research funding is split between a larger portfolio of lower risk grants, supporting more traditional and established research ideas, and a smaller portfolio of higher risk research. The key to building a high-risk public research portfolio is to implement a discordant selection scoring method where grant reviewers with the strongest opinions, both for and against, drive the selection process to avoid regression to means, and the research projects that are the most controversial get funded.

It is beyond the scope of this study to model and analyze the potential effects of introducing a barbell strategy for public research funding allocations, but we do see similar trends in technology innovation where motivated younger, less experienced founders are spearheading and accelerating disruptive innovation, e.g., founders of global companies such as PayPal, Tesla, Open AI, Cohere, Spotify, and Klarna.

## 5 Strengths and impacts of this study

The study suggests that policymakers and public decision-makers tend to obtain expert views from a small group of very experienced researchers (46) with an initiative to first-author research articles. Furthermore, we have not found any other studies of this kind where medical researchers have voiced their perceptions of research funding and the interface with public funders, and the study reveals perceptional nuances that were not previously identified.

First, the researchers are expected to communicate and disseminate their research to societal stakeholders to aid transfer of research knowledge and technology. One example of this is open access to research articles. The idea is that this offers the public sufficient information to advocate for changes to policies and research funding (47). From the perspectives of the old age and dementia researchers in this study, we have not found evidence of the intended transfer of knowledge or know-how per se or any other mutually and/or communicated objectives, but the researchers’ public communication is rather an intentional promotion of own their research and own research funding. Importantly, this communication also is between the more experienced researchers and the public funders. This is a limiting factor for younger researchers to market and attract public funding for own research ideas, unless this is aligned with the more experienced researchers’ interests. It is a takeaway from this study, that these inherent frictions to research communication should be considered reduced to avoid unwanted path dependencies in dementia research, 56% of the respondents believe too much research is spent on established research ideas, and a very high 14% of the respondents “prefer not to answer” that question.

Second, despite almost half (47%) of the researchers communicating with policymakers and public decision-makers, it is concerning that nine of 10 researchers do not think these officials have a very good understanding of the research. That is a signal of a communication breakdown, which would be interesting to examine more closely, both with respect to public health management and training of medical researchers.

Third, international public health faces a situation where dementia research, after 40 years, still offers no prevention or cure and continues to be chronically underfunded (7), medical and social costs are accelerating exponentially, particularly in high-income countries with an aging population. New and novel research ideas are needed to improve dementia research results. This study shows that dementia researchers are prepared to contribute to these societal needs. It is an untapped public health potential of optimization, nine of 10 researchers are willing to discuss the alignment of public health objectives and researcher objectives. This is underwritten by also nine of 10 dementia researchers reporting that new and novel research ideas are needed on their old age and dementia research area. The caveat is that these new and novel ideas are not only coming from the more experienced researchers who communicate with policymakers and public decision-makers today. This study and the transformative effects of new technologies and AI in medical research (48) suggest that it may be due time to consider implementing a structured communication platform between medical researchers, policymakers, and public funding decision-makers to allow communication between the parties. It is beyond the scope of this study to discuss the form and extent of such communication platforms.

## 6 Limitations

Research communication and research funding have been studied in detail from a public funding and institutional perspective. Earlier studies have shown that the drives and perceptions of public decision-makers and researchers are very different (5). To seek the dementia researchers’ perceptions and views we developed a new survey for this purpose. An extensive validation process was executed and described in the study provide transparency and possible repeating, but employing a novel survey questionnaire should be considered a limitation to the study. Care was taken with respect to balancing the risks of bias, ambiguity, over-representation, and fraud (20). Furthermore, the limitations of online surveys were considered, hereunder lack of follow-up questions, sharing of the survey with non-invited respondents, which can lead to over-representation, deliberate erroneous responses, and lower response rates and bias (28).

Response rate is a second limitation of this study. Numerous studies cover this topic and an average response rate for online surveys may be argued to be 36% (29, 31, 32). A low response rate may result in a non-representable survey population and introduce non-response bias, which increases uncertainty and unstable estimates. This can be expected for this study as it has been reported low response rates by respondents in an academic setting and of a professional population (29). Studies have shown that surveys with sample size below 500 can be reliable with a sample size of 20–25% (29, 33). The survey has 91 respondents and a response rate of 23.3%, and this was considered acceptable for the descriptive results pursued and for the exploratory study in a new area (49). Nevertheless, care should be taken to infer causal relationships from the data and results presented (30).

Differences in statistical significance between *p*-values of the mean (SD) and median (IQR) suggest that the data distribution could be skewed or have outliers, as this would affect means more than the median. A larger sample size in future research could offer different *p*-values and variabilities.

Funding own research is necessary for many researchers; care should therefore also be made interpreting the absolute reported numbers. The answers could reflect the responders’ current ability to fund their own research, where successful funders answer more favorably to the current public funding model and vice versa, introducing status quo bias (50, 51).

The H-index is an important scientometric indicator for researchers in sciences and medicine (52), but it can be influenced and the reported values can vary (53). This study is based on self-reported H-index values, which can be considered an additional uncertainty and should be considered when interpreting the study.

## 7 Future research

Future research should endeavor to repeat the study with a larger sample size and/or in other medical specialties to develop causal effects, which can help researchers fundraise research more effectively. The authors will make the questionnaire used in this study available upon request.

Public funders require researchers to communicate their research (54). This study suggests that dementia researchers also communicate with public funders on more general topics to help fundraising future research, but we cannot find evidence in literature or this study that such communication with public funders causes more funding. Policymaking and public decision-making are complicated and complex (14). Given the results of this study, it can be valuable to explore whether the rationale of actions and expected effects (program theory of change) (55) can be compared/aligned for medical researchers, policymakers, and public research decision-makers, rather than assuming intuitive resulting effects (56), i.e., the tendency to interpret results surrendering to hindsight bias. This study suggests that the relationships among inputs, activities, outcomes, and impacts in the research communication and the funding thereof may not function as anticipated, and despite ongoing research communication, the researchers perceive a significant lack of research understanding by the public funders.

Future research could therefore separate how to communicate, what to communicate, and why to communicate, to clarify the different roles and interests at play in the researcher–policymaker–public decision-maker interface—and study its anticipatory effects compared with empirical effects. This can be particularly important with the current transformation of innovation and technology through the implementation of AI in a wide variety of workstreams throughout society.

In the discussion, we offered an alternative public funding strategy to inspire more new and novel research, through a barbell strategy, where, e.g., disruptive discordant scoring methods for novel research ideas can be useful. Future research could explore how such discordant scoring methods can be designed and implemented effectively to increase funding to novel research ideas outside the established dementia research hypotheses.

## 8 Conclusion

In old age and dementia research, communication with policymakers and public decision-makers is performed by slightly less than half (43%) of the dementia researchers. These communicating researchers have significantly more research experience and have a higher H-index than the researchers who do not communicate with these public officials. HOWEVER, WE SHOULD recognize that the individuals may not be well equipped to communicate or translate the totality of brain research in a manner that allows them to construct effective and equitable public health policies. The communication is mostly about the importance of their own research and with the purpose of funding their own research projects going forward. 95% of dementia researchers believe that new and novel research ideas are needed to help society prevent and cure one of the largest public health challenges in our time. However, funding decisions do not always reward innovation. A research communication model to transfer knowledge from research and complement policymaking has failed and the European Commission, and other national governments’ drive to ensure that research and innovation activities make substantive contributions to societal wellbeing by securing social support and facilitating researchers’ guidance to society is at stake. The introduction of research work packages requiring communication by senior and more junior researchers, hereunder development of research narratives, could offer more representative and attractive research information for public decision-making processes. Timely and structured dialog between policymakers, public decision-makers, and dementia researchers can provide insights with respect to political “window(s) of opportunity” for change, thereby guiding the researchers to developing more appropriate and effective research communication. A revised public funding strategy with public research grants based on discordant scoring can promote more new and novel research.

## Data Availability

The raw data supporting the conclusions of this article will be made available by the authors, without undue reservation.

## Footnotes

^1^ www.uio.no/english/services/it/adm-services/nettskjema/
